# Machine learning reveals singing rhythms of male Pacific field crickets are clock controlled

**DOI:** 10.1093/beheco/arad098

**Published:** 2023-12-23

**Authors:** Mary L Westwood, Quentin Geissmann, Aidan J O’Donnell, Jack Rayner, Will Schneider, Marlene Zuk, Nathan W Bailey, Sarah E Reece

**Affiliations:** Astrophysics, Department of Physics, University of Oxford, Oxford, UK; Center for Quantitative Genetics and Genomics, Aarhus University, Aarhus, Denmark; Institute of Ecology and Evolution, School of Biological Sciences, University of Edinburgh, Edinburgh, UK; Department of Biology, University of Maryland, College Park, College Park, MD, USA; School of Natural Sciences, Bangor University, Bangor, UK; Department of Ecology, Evolution and Behavior, University of Minnesota, St. Paul, MN, USA; Centre for Biological Diversity, School of Biology, University of St Andrews, St Andrews, UK; Institute of Ecology and Evolution, School of Biological Sciences, University of Edinburgh, Edinburgh, UK; Institute of Immunology and Infection Research, School of Biological Sciences, University of Edinburgh, Edinburgh, UK

**Keywords:** bioacoustics, chronobiology, circadian rhythms, machine learning, Rethomics, sexual signals, stridulation, temperature compensation, Tempaural

## Abstract

Circadian rhythms are ubiquitous in nature and endogenous circadian clocks drive the daily expression of many fitness-related behaviors. However, little is known about whether such traits are targets of selection imposed by natural enemies. In Hawaiian populations of the nocturnally active Pacific field cricket (*Teleogryllus oceanicus)*, males sing to attract mates, yet sexually selected singing rhythms are also subject to natural selection from the acoustically orienting and deadly parasitoid fly, *Ormia ochracea*. Here, we use *T. oceanicus* to test whether singing rhythms are endogenous and scheduled by circadian clocks, making them possible targets of selection imposed by flies. We also develop a novel audio-to-circadian analysis pipeline, capable of extracting useful parameters from which to train machine learning algorithms and process large quantities of audio data. Singing rhythms fulfilled all criteria for endogenous circadian clock control, including being driven by photoschedule, self-sustained periodicity of approximately 24 h, and being robust to variation in temperature. Furthermore, singing rhythms varied across individuals, which might suggest genetic variation on which natural and sexual selection pressures can act. Sexual signals and ornaments are well-known targets of selection by natural enemies, but our findings indicate that the circadian timing of those traits’ expression may also determine fitness.

## Teaser

Using machine learning, we found that male Pacific field crickets singing (which makes them obvious to mates, parasites, and predators) has a daily, biological rhythm.

## INTRODUCTION

The daily rotation of the Earth causes predictable cycles of day and night, which nearly all life has evolved to cope with. Circadian clocks (i.e., daily, biological timekeepers) are ubiquitous and allow organisms to schedule activities, from gene expression to physiologies to behaviors, according to the time-of-day they are best undertaken (Johnson et al. 2004). Most research on circadian rhythms has focused on uncovering the genes and molecular pathways involved in the workings of circadian clocks. However, there is increasing interest in the evolution and ecology of circadian rhythms—particularly, in how rhythms affect survival and reproduction (Greives et al. 2015; Hau et al. 2017; Rubin et al. 2017; Westwood et al. 2019; Hozer et al. 2020). Overt and rhythmic sexual signals provide an opportunity to examine these questions as they often put the signaler at risk of predation and/or parasitism, and so, are subject to natural selection as well as sexual selection. However, whether such rhythms are simply direct responses to a change in environment light/dark levels, or scheduled by an endogenous circadian clock, is poorly understood.

A well-studied case in which a rhythmic mating behavior is subject to both natural and sexual selection concerns the Pacific field cricket, *Teleogryllus oceanicus*. These crickets are introduced in Hawaii, where they are subject to the lethal, acoustically orienting parasitoid fly *Ormia ochracea*. Strong natural selection against “normal-wing” singing males has led to the evolutionary spread of distinct male forms which silence or reduce their song, protecting them against the fly (e.g., flatwing, curlywing, small-wing, and purring phenotypes) (Zuk et al. 2006; Pascoal et al. 2014; Tinghitella et al. 2018; Rayner et al. 2019). Pre-existing satellite behavior (i.e., employing a silent strategy whilst intercepting females attracted to singing males) likely facilitated the spread of these mostly silent/altered wing morphs throughout the Hawaiian Islands (Bailey et al. 2007). However, the loud, long-range calling song is much more conspicuous to females, which also show preference for normal-wing song. Thus, conferring normal-wing males a mating advantage and explaining, at least in part, their persistence in the wild (Bailey and Zuk 2008; Tinghitella and Zuk 2009; Tinghitella et al. 2021).

While the singing of normal-wing males renders them vulnerable to parasitism, *O. ochracea* may not be positively phonotactic (i.e., attracted to sound) throughout the entirety of the night. Indeed, multiple studies suggest *O. ochracea* phonotaxis to *T. oceanicus* song peaks around dusk (Cade 1979; Kolluru 1999) and trails off prior to sunrise. However, observations indicate Hawaiian *T. oceanicus* curb singing around both dawn and dusk (Zuk et al. 1993) (compared to unparasitized, ancestral populations), though notably Kolluru (1999) found Hawaiian *T. oceanicus* activity peaks only at dusk, coinciding with the time of greatest fly phonotaxis. Regardless of the precise timing of fly phonotaxis, whether and how a restricted singing window can evolve depends on how it is controlled. If the onset of the singing rhythm is a direct response to experiencing dusk (i.e., behavioral plasticity) then delaying singing requires either the evolution of the usage of a different cue such as even lower light intensity, or the evolution of a delay between cue and response. This could occur if selection acts on existing genetic variation for photosensitivity or lag in response to light intensity. In contrast, if the singing rhythm is controlled by a circadian clock (Loher and Orsak 1985) how singing behavior responds to clock outputs could change. Because clocks and their outputs control much of an organism’s physiology and behavior (e.g., >80% and > 40% of protein-coding genes show daily, rhythmic expression in male baboons and mice, respectively; Zhang et al. 2014; Mure et al. 2018), alterations to clock mechanics may be constrained if singing is a cue for, or a tightly linked aspect of traits that have to occur in advance of mating. For example, if spermatophore maturation precedes the onset of singing by a fixed amount of time, singing onset may be temporally constrained (McFarlane 1968; Loher 1974). Under such scenarios, the extrinsic consequences of singing (e.g., parasitism risk and mate attraction) trade off with each other as well as with the intrinsic consequences (e.g., readying a spermatophore). Further complexity occurs when closely related species share a common landscape. For example, crickets of the genus *Laupala* (sympatric species *L. cerasina* and *L. paranigra*) exhibit significant daily temporal differences in singing (and thus mating), which likely reduces interspecific acoustic interference (Danley et al. 2007).

Understanding how singing rhythms can evolve requires knowledge of the extent of their circadian regulation, their sensitivity to variation in abiotic conditions (such as temperature), and their variation between individuals within a population. Research on circadian rhythms in crickets has largely mirrored that of chronobiology, with early work focusing on the ecology of rhythms (Loher and Edson 1973; Loher 1974, 1979; Loher and Wiedenmann 1981; Loher and Orsak 1985) and a subsequent shift in focus toward determining molecular clock mechanisms (Lupien et al. 2003; Moriyama et al. 2008, 2009, 2012; Uryu et al. 2013). Knowledge of how molecular clocks operate opens the door toward using this information to answer questions pertaining to the evolutionary ecology of circadian rhythms, particularly how circadian rhythms govern interactions between individuals (e.g., males and females, predators and prey, and hosts and parasites).

Early studies pertaining to circadian rhythmicity in *T. oceanicus* singing address some, but not all, requirements for a rhythm to be deemed circadian (Loher and Orsak 1985). Rhythms are deemed under the control of an endogenous circadian oscillator when they meet all four of the following requirements: (1) the duration of the rhythm has a “period” of approximately 24 h, (2) the rhythm persists (“free-runs”) under constant environmental conditions, (3) the timing (i.e., the “phase,” as quantified by the onset, peak, or offset) of the rhythm is set (“entrained”) by a periodic environmental time-cue (“Zeitgeber”), and (4) the pace of the clock is unaffected by a biologically realistic range of temperatures (“temperature compensation”), which is usually examined under free-running conditions. Zeitgeber time (ZT), which we use to describe phase markers, is the timing of an output or behavior in relation to the Zeitgeber, with ZT0 indicating the start of the light (or subjective light) phase. For example, if a behavior occurred 2 h into the light phase, the timing of that behavior is described as ZT2.

Verifying these characteristics in the form of behavioral assays is achieved by observing rhythmicity for multiple consecutive days. A standard method in chronobiology is to test for entrainment (i.e., aligning a rhythm to a Zeitgeber) by changing conditions from a standard photoschedule (12 h light: 12 h dark; LD) to a reversed photoschedule (12 h dark: 12 h light; DL), in case rhythms in rearing conditions happen to be driven by something else (since a change from LD to DL is the greatest perturbation that can be made). Further, observing rhythms in either constant light (LL) or constant darkness (DD) while holding all other variables constant allows us to determine the free running period of the rhythm, and observing rhythmicity under various temperatures allows us to examine its stability over a range of temperatures. Without verification of each of these characteristics, an observed rhythm may simply be the direct response of an organism to a change in the external environment and not the product of a cellular autonomous circadian oscillator. For example, Tan and Robillard (2021) observed some time-of-day variation in singing activity across 11 cricket species but were unable to parameterize rhythms or determine whether an endogenous oscillator is involved.

Here, we ask whether the singing rhythm of *T. oceanicus* is circadian (i.e., whether they meet the four conditions stated above), and we assess individual variation in rhythmic parameters. To do this, we develop a novel audio-data-to-circadian analysis pipeline for the extraction (via “Tempaural,” a bespoke R package we implement in the “Rethomics” analysis framework), processing (through machine learning), and analysis of around-the-clock continuous audio data. We then deploy our method to analyze data from three experiments, revealing that singing rhythms are under endogenous circadian clock control, driven by photoschedule, and robust to variation in temperature. Furthermore, individual variation underlies differences in parameters that characterize singing rhythms.

## MATERIALS AND METHODS

### Animals, rearing, and experimental conditions

Experimental subjects were taken from laboratory stock populations established in 2012 from females collected in Lai’e, Hawaii (Schneider et al. 2018). At the time of establishment, approximately 50% of males in the population expressed the flatwing phenotype. For the purpose of this experiment, we excluded flatwing males and hereafter, “adult male” refers to the normal-wing phenotype. We housed both stock and experimental animals in 9 L plastic boxes with egg cartons for shelter and fed Burgess™ Excel Junior and Dwarf rabbit pellets with water available ad libitum. Rearing conditions consisted of a LD photoschedule (lights-on at 06:00 UTC and lights-off at 18:00 UTC), and temperature was maintained at 25 °C. To house crickets during the experiment, we used either Panasonic MIR-154-PE Cooled Incubators or LEEC SFC3C R/H Ultrasonic Humidity Cabinets. Males in each experiment were within 3 days post-eclosion and physically and acoustically isolated from all other males for the duration of the recordings. Acoustic isolation was achieved by housing each cricket alone in its own incubator. During experiments, all recordings were made from individual crickets with food and water ad libitum and egg carton for shelter.

### Experimental designs

We conducted three experiments to test whether *T. oceanicus* singing rhythms fulfill the criteria for control by an endogenous clock. Our first experiment (experiment 1) was designed to verify singing is nocturnal and characterize its basic daily patterns. We then carried out two further experiments to test whether singing rhythms persist in the absence of a time-of-day cue, and if rhythms are robust to temperature variation under both constant (experiment 2) and entrainment (experiment 3) conditions. Specifically, experiment 2 tests whether the period of a given rhythm is maintained over a range of temperatures, and experiment 3 probes whether entrainment (when e.g., the onset, peak, and offset of a rhythm occurs in relation to the Zeitgeber) occurs in a manner independent of temperature. We quantify onset, peak, and offset using the widely used chronobiology software ClockLab (ActiMetrics, Wilmette, IL, USA). As is standard in chronobiology, we define onset and offset as the ZT in which 20% of the activity peak is reached pre- and post-peak, respectively (and peak is defined as the maxima of activity in a 24-h period).

#### Experiment 1: Characterizing the singing effort and timing of singing across days

**Figure 1:**
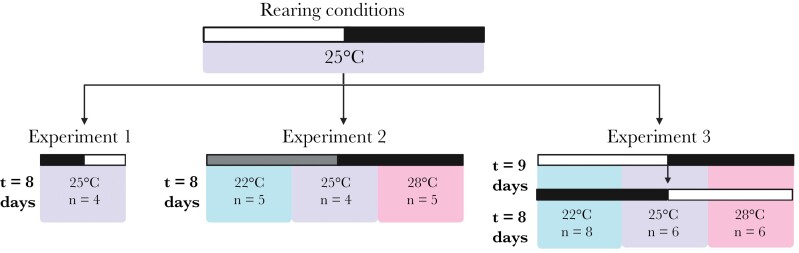
Rearing and experimental conditions. Individuals were removed from rearing conditions and placed into incubators where they were recorded for at least 7 days per photoschedule regime. Crickets in experiment 1 were recorded under photoperiod-reversed conditions (DL) relative to rearing conditions, crickets in experiment 2 were recorded in constant darkness (DD), and crickets in experiment 3 were recorded under standard (LD) and then photoperiod-reversed conditions (DL). White bars indicate the light phase of a photoperiod and black bars indicate the dark phase of a photoperiod; each bar represents 12 h. As experiment 2 was performed in constant darkness, a gray bar indicates which part of the photoschedule was previously light and we use the terms “subjective day” to refer to the gray portion of the photoschedule, and subjective night to refer to the black portion of the photoschedule. Temperature for each group is indicated by color (blue = 22 °C, purple = 25 °C, and pink = 28 °C).

Adult males (*n* = 4) were recorded continuously for at least 8 days under photoperiod-reversed (DL) conditions relative to standard rearing conditions, and constant temperature (25 °C) (Figure 1). Recording was performed under DL conditions to ensure singing rhythms are driven by lighting rhythms (i.e., that singing will entrain to the new, reversed photoperiod). We used these audio recordings to estimate the period of singing under predictable environmental conditions, to determine the singing effort (i.e., the average number of minutes per day that a cricket sang at least once) and the proportion of time spent singing each day, and to determine how much singing occurs during the light versus dark phase.

#### Experiment 2: Fundamental circadian properties

Adult males (*n* = 14) were haphazardly assigned to one of three temperature treatment groups (22 °C, *n* = 5; 25 °C, *n* = 4; 28 °C, *n* = 5) (see Figure 1) all in constant darkness (DD) and recorded continuously for at least 8 days. Audio recordings from these crickets were used to test whether singing rhythms free run (i.e., the rhythm persists in the absence of rhythmic environmental cues) and are temperature compensated (i.e., the rhythm maintains an approximately 24 h period despite the different temperatures).

#### Experiment 3: Temperature compensation under entrainment

To further probe for temperature compensation we tested whether the process of entrainment in response to a change in photoschedule is temperature compensated. Adult males (*n* = 17) were haphazardly assigned to one of three treatment groups: 22 °C (*n* = 8), 25 °C (*n* = 6), or 28 °C (*n* = 8) (see Figure 1). We recorded each cricket under standard LD conditions, then at lights on (Zeitgeber Time, ZT0) on day 9, the incubators had been preprogramed to switch to photoperiod-reversed (DL) conditions, and we recorded each cricket for a further 8 days. By reversing (“phase-shifting”) the photoschedule during the experiment, we tested whether and how crickets are able to entrain to an altered photoperiod, and whether the process of entrainment is temperature compensated. Entrainment is the process by which the period of the circadian clock aligns with the period of a Zeitgeber; that is, singing re-aligning its rhythmicity with the reversed photoperiod. Day-to-day temperatures vary in nature and so the process of entrainment (e.g., in response to seasonal photoperiods) itself should be temperature compensated; manifesting as no observable difference in the manner rhythms shift between temperature treatment groups during adjustment from LD to DL. Additionally, we calculated the singing effort (as described for experiment 1) as one indicator of variation amongst individuals.

Prior to circadian analysis for all experiments, we removed the first 72 h from each individual’s dataset to allow for acclimation to experimental conditions. Further, we removed the first 4 days post-photoperiod-reversal to allow for transient cycles (i.e., the time necessary for a rhythm to reach a stable phase-relationship with the central circadian pacemaker) (Pittendrigh and Daan 1976). The resulting days of recordings (minus the days stated above) were the only ones used in analyses. We chose to record on days that were ultimately discarded to lessen perturbation to the crickets and their environments.

### Audio recordings

We collected continuous audio data using recorders (Sony™ ICD-UX560 Digital Voice Recorders equipped with Integral™ Micro Secure Digital eXtended Capacity cards) set to a sampling rate of 44.1 kHz and a 16-bit resolution (stereo MP3 file format). Recorders were adhered to the inside of each plastic box and fitted with an external power supply cord. We transferred audio files to external hard drives (Western Digital 4TB Elements Portable Hard Drive—USB 3.0) prior to analysis on a personal computer (2017 Apple MacBook Pro using macOS Catalina) and the University of Edinburgh’s high performance computing cluster.

### Identifying singing and characteristics of singing rhythms

Crickets produce acoustic signals by rhythmically opening and closing their forewings, rubbing the scraper and file together (i.e., “stridulation”) (Pfau and Koch 1994) and bouts of singing generally last from several seconds to minutes (Schneider et al. 2018). Calling song produced by normal-wing *T. oceanicus* has a dominant frequency between 4-5 kHz and is characterized by a long chirp followed by a series of short chirps (Balakrishnan and Pollack 1996). Given the inter-individual variability in signal (pitch, amplitude, sparsity of the chirps, etc) and noise (electromagnetic noise, incubator fans), we could not trivially (e.g., thresholding the signal amplitude in the dominant frequency band) and accurately detect songs. Instead, we described multiple spectral properties of *T. oceanicus* song (see Supplementary Appendix Table I), and trained a random forest model, using k-fold cross validation, to predict whether a cricket sang during each consecutive 60s audio clip (hereafter referred to as a “clip”) throughout its entire recording window (8–18 days per individual, depending on the experiment). Using a random forest model allowed us to non-linearly combine multiple descriptors and approximate the best classification criteria, and to efficiently process the audio files that exceeded 1TB (>12,000 h) across all experiments. To facilitate and standardize the analysis of this large amount of data, we developed “Tempaural,” an R package that interfaces bioacoustic data with the Rethomics framework. Tempaural is freely available at https://github.com/rethomics/tempaural.

To generate our random forest model (Breiman 2001), we first randomly extracted 557 × 60 s clips from seven representative crickets spanning three different experimental conditions (i.e., LEEC or Panasonic incubator; 22 °C, 25 °C, or 28 °C) and length of time spent in an incubator (8–18 days) (see experimental designs), thus accounting for incubator type, temperature, and cricket age in the training and validation of the model. Saved clips were tagged with a random string to ensure anonymization and randomization. We then manually listened to all clips and classified them as “singing” as a binary response variable (the cricket was heard singing at least once during the clip, this includes short chirps lasting ~1 s; singing = 1) or “background” (the cricket was not heard singing during the clip; singing = 0).

Next, we initially extracted 19 audio features (e.g., descriptive statistics of the frequency in the 3–6 kHz spectrum) from each clip using the bioacoustics R package “Seewave” (Sueur et al. 2008) (see Supplementary Appendix Table I for the full list of features). These predictors were iteratively pared down to five which returned a high level of accuracy on the training set (Supplementary Appendix Table I). We used the Caret package in R to split the data into training and validation sets (75% and 25%, respectively) (Kuhn 2008) and trained a set of models (classification and regression tree, k-Nearest Neighbors, and random forest) using *k*-fold cross validation (*k* = 10) of which the random forest model performed best (accuracy = 0.978, κ = 0.918). We estimated the performance of the model on our validation set, which returned a very high level of accuracy (accuracy = 0.985, confidence interval [CI] [0.949, 0.998], κ = 0.948) (Supplementary Appendix Tables II–III and Figure I). We then applied this model to score on all consecutive 60 s clips as “singing” (1) or “not singing” (0). We averaged the values across clips from the simultaneous recordings of individuals in the same treatment groups to generate a continuously distributed variable for analysis and presentation of some results, which is shown as the legend scale (from 0 to 1) and can be interpreted as the mean “singing” for a given minute. This averaging can be seen on Figure 3B–D and Figure 4A–C.

### Statistical and circadian analysis

We used R v4.0.1 (R Core Team 2021) for all analyses, except for the derivation of phase markers (onset, peak, and offset) which we obtained using ClockLab software (ActiMetrics, Wilmette, IL, USA).

By examining double-plotted actograms for each cricket we carried out initial visual inspection of singing for the duration of each experiment. As “singing” was quantified as either a “0” or “1” for each minute, the legend scale (from 0 to 1) on Figure 3B–D and Figure 4 can be interpreted as the mean “singing.” Double-plotted actograms are a standard visualization approach; they are “double-plotted,” as the x-axis shows 48 h (instead of 24 h), and the second 24 h on each row (i.e., hours 24–48) is re-plotted as the first 24 h on the subsequent row (i.e., below hours 0–23). Time (in “Days”) is plotted on the y-axis, and “Days” corresponds to the first 24 h plotted on each row. The black, white, or gray bars at the top of plot indicate dark, light, subjective light, or subject dark phases (as explained in the legend of the schematic in Figure 1). If circadian, the behavior or output should occur each day at the same approximate time in relation to the Zeitgeber (or, subjective Zeitgeber). If the timing of the Zeitgeber is perturbed, the rhythm should re-entrain to the new timing by either phase advancing (i.e., moving earlier) or phase delaying (i.e., moving later) to regain a stable phase relationship to the Zeitgeber (i.e., when a rhythm occurs in relation to the Zeitgeber).

We excluded 1 cricket from experiment 2 at 25 °C due to no singing, and we excluded two crickets from experiment 3 at 28 °C (one for death, and the other for not singing). Recording equipment failure resulted in the loss of ~24 h of data from two crickets in experiment 2 and ~12 h of data from 1 cricket in experiment 3, though these crickets were retained in the final dataset because the remainder of their recordings were unaffected. Not every individual sang every day (or to a degree in which onset, peak, and offset were detectable); these individuals were retained in the dataset, and onset, peak, and offset were calculated only for those days in which they could be confidently estimated.

Free-running and entrained period estimates (i.e., periods under constant environmental conditions such as in experiment 2, and periods under entrained environmental conditions such as in experiments 1 and 3, respectively) were calculated using Lomb-Scargle (LS) periodograms (Ruf 1999) via the Rethomics workflow (Geissmann et al. 2019). LS periodograms calculate a Fourier-like power spectrum from which the period of oscillation can be determined and significance (α = 0.05, i.e., the data are rhythmic) may be tested (Ruf 1999). Mean singing activity, free-running periods (FRP), and entrained periods were compared using *t*-tests and Kruskal–Wallis tests (Kruskal and Wallis 1952). Circular data (onset, peak, and offset) were modeled using Bayesian projected normal circular regression models compared by the “Watanabe-Akaike information criterion” (WAIC) (Watanabe and Opper 2010) using the R package “bpnreg” (v. 2.0.2). A change in 2 WAIC (ΔWAIC = 2) was chosen to select competitive models. The most parsimonious of the competitive models was chosen for interpretation, and coefficients were considered significant if the high posterior density estimates varied from zero (Cremers et al. 2018). Finally, individual variation in phase markers was estimated via angular variances (*V*_*m*_) (Jammalamadaka et al. 2001) also using the R package “bpnreg.”

Data and code are available on DRYAD (https://datadryad.org/stash/dataset/doi:10.5061/dryad.z08kprrkb) (Westwood).

## RESULTS

### Experiment 1: Temporal characterization

**Figure 2:**
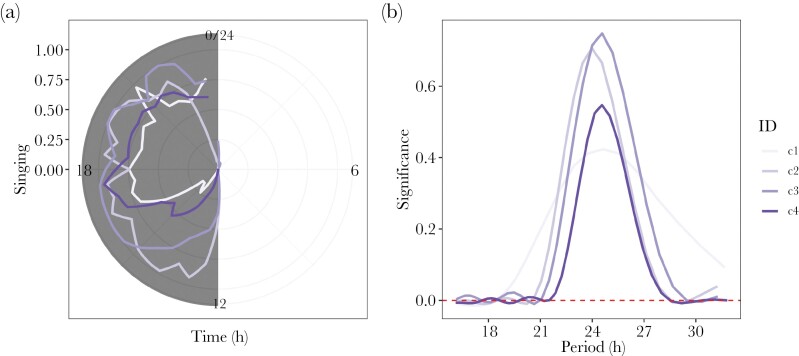
(A) Group polar coordinate plot for photoperiod reversed crickets. Purple lines represent singing activity averaged and wrapped across 24 h for each individual. Shaded gray and white areas indicate dark and light phases as experienced during the experiment, respectively. Polar coordinates (0/24, 6, 12, and 18) represent time (ZT; in hours), and distance from the center of the plot (indicated on the upper left quartile of the leftmost plot) illustrates average singing value (0 = no singing and 1 = singing recorded in at least part of a clip) for each cricket at a given 30-min window across consecutive days of recording. (B) LS periodograms for individual crickets under entrained, photoperiod reversed conditions. Period estimate (in hours) is shown on the x-axis and the power of the period estimates is shown on the y-axis. The horizontal, red dashed line is the cut off for a significant period estimate (α = 0.05, i.e., rhythmic). The period estimate with the highest power for each individual was accepted for further analysis. For A and B, colors represent *n* = 4 individual crickets (c1-c4).

When placed under photoperiod reversed conditions at constant temperature, crickets sing significantly more in the dark than the light phase (*t* = 5.35, *P* = 0.013, df = 3, 95% C.I. [1146.46, 4494.54]; Figure 2A). Indeed, crickets sing, on average, at least once per minute during 68% of the dark phase of the experiment (mean = 0.68 ± 0.18 SD) in contrast to only 1% of the light phase (mean = 0.01 ± 0.01 SD). In total, the most reserved cricket sang at some point during each minute for as little as 9.32 h per day (singing effort = 9.32 ± 0.00021 h SE) compared to the most vociferous cricket which sang at least once per minute for upwards of 12.7 h per day (singing effort = 12.7 ± 0.00022 h SE). Onset of singing began about an hour into the dark phase (mean onset = ZT13.06 ± 1.67 h SD), peaked nearly 5 h later (mean peak = ZT17.61 ± 1.95 h SD) and tapered off just before the start of the light phase (mean offset = ZT23.91 ± 2.46 h). Further, the entrained LS period estimate was close to 24 h (mean period = 24.4 h, SE = ± 0.15 Figure 2B). These results show, as expected, males sing overwhelmingly during the dark phase and vary greatly in how much they sing on average per day.

### Experiment 2: Fundamental circadian properties

**Figure 3:**
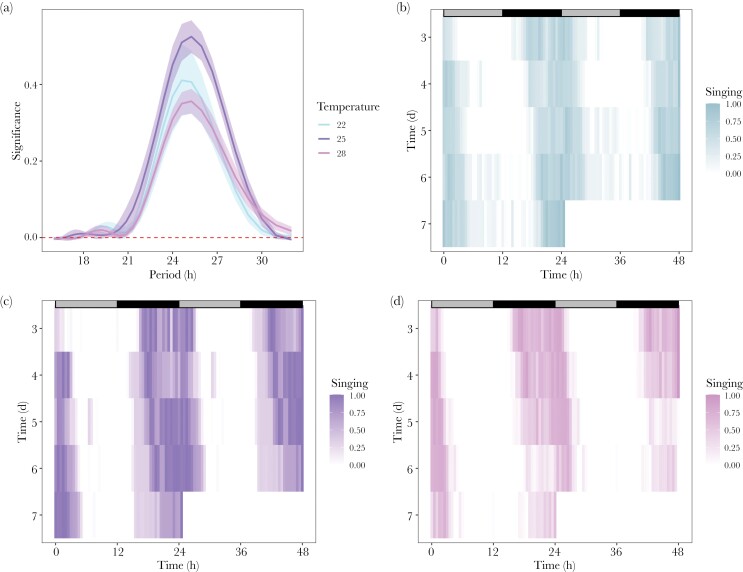
(A) LS periodograms for each temperature treatment group under free-running conditions. Period estimate (in hours) is shown on the x-axis and the power of the period estimates is shown on the y-axis. The horizontal, red dashed line is the cut off for a significant period estimate (α = 0.05, i.e., rhythmic). The period estimate with the highest power for each individual was accepted for further analysis. (B–D) Double-plotted (i.e., 48 h) actograms averaged across all individuals within each temperature treatment (B, 22 °C = blue, C, 25 °C = purple, and D, 28 °C = pink) showing singing rhythms under free-running conditions (constant dark). Subjective light and dark phases are indicated by gray and black bars (respectively) situated at the top of each plot. Time in days is shown on the y-axis and time in hours is on the x-axis. Legends indicate singing as depth of color. Days 0–2 are removed to allow for acclimation to experimental conditions.

Free-running periods (FRP) are characteristically close to, but never exactly, 24 h (Pittendrigh and Daan 1976). In keeping with this, FRP for each temperature group is slightly longer than 24 h (mean ± SE: 22 °C = 25.0 ± 0.16; 25 °C = 25.2 ± 0.01 28 °C = 25.1 ± 0.13; Figure 3A; Supplementary Appendix Figure II) and does not differ significantly between temperature groups (Kruskal–Wallis, χ^2^ = 1.6, *P* = 0.45, df = 2) (Figure 3A), giving an overall FRP of 25.1 ± 0.08 SE. Because FRPs are not precisely 24 h, circadian rhythms drift over successive days while under constant conditions. For *T. oceanicus,* the elongated FRP delays the onset of singing each day, pushing onset further into subjective night and offset into subjective day (Figure 3B–D; see Supplementary Appendix Figure II for individual LS periodograms). These results show that singing rhythms in *T. oceanicus* are endogenous, close to 24 h and temperature-compensated.

### Experiment 3: Temperature compensation under entrainment

**Figure 4:**
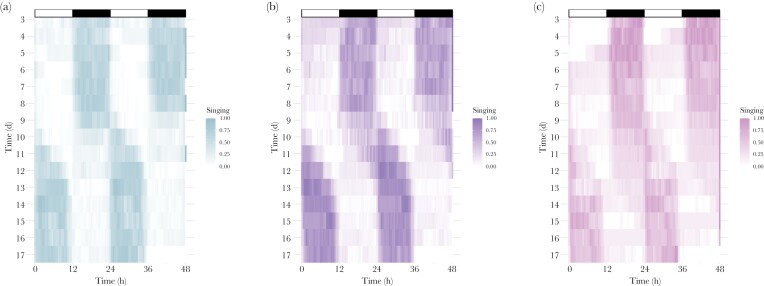
(A–C) Average double-plotted actograms for each of three temperature treatments (A. 22 °C = blue, B. 25 °C = purple, and C. 28 °C = pink) showing singing rhythms under entrained conditions (LD, white and black bars on top of plot) and following photoperiod-reversal at ZT0 on day 9 (red arrows). Time in days is shown on the y-axis and time in hours is on the x-axis. Legends indicate singing as depth of color. Days 0–2 are removed to allow for acclimation to experimental conditions.

Across all temperature treatment groups, crickets sing during the dark phase under standard lighting conditions (days 3–8, Figure 4) and in photoperiod reversed conditions, following several days of adjustment (days 13–17, Figure 4). Specifically, upon photoperiod reversal on day 9, crickets begin to shift singing patterns (delaying onset and offset) until re-aligned with their new photoschedule (days 13–17, Figure 4).

**Figure 5:**
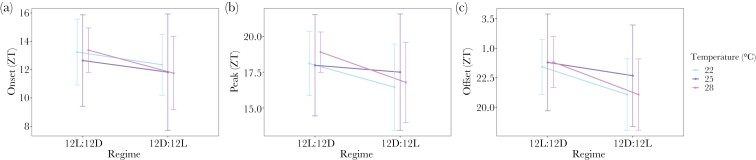
Mean phase markers (y-axis; A. Onset, B. Peak, and C. Offset in Zeitgeber time (ZT); mean ± SD) for each temperature treatment (legend; 22 °C = blue, 25 °C = purple, and 28 °C = pink) across both photoschedule regimes (x-axis; LD and DL).

The process of entrainment and the resulting rhythms follow similar patterns across temperatures (Figure 5; Tables 1–2). The most parsimonious model for onset and peak included both regime (i.e., the photoschedule regime as outlined in Figure 1) and temperature as main effects (ΔWAIC = 0 and 1.30, respectively; Table 1). However, because the high posterior density for each temperature treatment contained 0 for both phase markers, but not for regime, we interpret regime as the main driver in any observed variation in both onset and phase. Specifically, upon phase shift onset and peak were phase advanced by ~1 h and ~1 h 18 min, each (mean = 0.97 ± 0.25 h SD and mean = 1.30 ± 0.30 SD for onset and peak, respectively) across the remainder of the experiment post-photoperiod reversal. Similarly, the most parsimonious model for offset included only regime (ΔWAIC = 0; Table 1) which resulted in a phase advance of just over 2 h upon phase shift (mean = 2.08 ± 0.34 h SD). Overall, we find that while temperature may increase model fit for some phase markers, it does not significantly contribute to explaining any observed variation. Also, we found moderate evidence for a phase advance (~2 h) in each of the three phase markers upon photoperiod reversal.

**Table 1:** Phase markers (“onset,” “peak,” and “offset”; “response”) are modeled by “regime” (LD or DL) and “temperature” (22 °C, 25 °C, and 28 °C) (“covariates”) as determined in experiment 3

Response	Covariates	WAIC	pWAIC	ΔWAIC	lppd	WAIC*w*
Onset	Regime + temperature	425.14	11.01	0.00	−201.56	0.50
	Regime × temperature	425.32	17.43	0.18	−195.23	0.46
	Regime	430.60	5.52	5.46	−209.78	0.03
	Temperature	438.89	7.87	13.75	−211.58	0.00
	Null	444.93	2.66	19.79	−219.81	0.00
Peak	Regime × temperature	490.57	14.07	0.00	−231.22	0.57
	Regime + temperature	491.87	8.84	1.30	−237.10	0.30
	Regime	493.62	4.43	3.05	−242.38	0.12
	Temperature	516.65	6.53	26.07	−251.79	0.00
	Null	517.85	2.15	27.28	−256.77	0.00
Offset	Regime	517.68	5.06	0.00	−253.79	0.49
	Regime + temperature	518.12	9.58	0.43	−249.48	0.40
	Regime × temperature	520.71	14.86	3.02	−245.50	0.11
	Null	551.40	2.26	33.71	−273.43	0.00
	Temperature	552.39	6.82	34.70	−269.38	0.00

WAIC, estimated number of parameters (pWAIC), ΔWAIC (WAIC_*model*_—WAIC_*min model*_), log pointwise predictive density (lppd) and WAIC *w* (WAIC weight) are shown for each model. Models are ordered in descending fit (best-fitting model at the top for each response).

**Table 2:** Phase markers (“onset,” “peak,” and “offset,” mean ZT ± S.D.), angular variances (*V*_*m*_ onset and *V*_*m*_ offset), and singing effort per day (“singing effort,” the average number of minutes per day that a cricket sang at least once; mean h ± SD) by temperature (“temp,” °C) and regime (LD and DL) as determined in experiment 3

Regime	Temp (°C)	Onset (ZT)	Peak (ZT)	Offset (ZT)	*V*_*m*_ Onset	*V*_*m*_ Peak	*V*_*m*_ Offset	Singing effort
LD	22	13.24 ± 2.33	18.11 ± 2.24	23.42 ± 2.33	0.34	0.32	0.34	8.16 ± 4.85
	25	12.64 ± 3.22	17.98 ± 3.54	23.80 ± 4.11	0.60	0.70	0.87	9.25 ± 4.63
	28	13.38 ± 1.57	18.90 ± 1.41	23.82 ± 2.16	0.17	0.13	0.30	9.31 ± 3.99
DL	22	12.34 ± 2.15	16.45 ± 3.01	21.07 ± 3.03	0.29	0.53	0.54	7.22 ± 2.90
	25	11.82 ± 4.11	17.50 ± 4.06	22.67 ± 4.30	0.88	0.86	0.94	7.94 ± 2.86
	28	11.75 ± 2.59	16.79 ± 2.80	21.07 ± 3.02	0.41	0.47	0.54	8.08 ± 4.26

Finally, average *T. oceanicus* singing rhythms under entrained conditions (i.e., for both LD and DL lighting regimes) are characterized by a period estimate of 24.72 ± 0.16 h SE, a mean onset of ZT12.48 (± 2.77 h SD), peaking at ~ZT17.47 (± 3.07 h SD), and mean offset of ZT22.37 (± 3.39 h SD). These parameters varied between individuals, with the angular variances (*V*_*m*_; Fisher 1995) ranging from 0.17–0.88 for the onset, 0.13–0.86 for the peak, and 0.30–0.94 for the offset (Table 2) (Batschelet 1981; Jammalamadaka et al. 2001). Further, singing effort over a circadian cycle averaged at 8.26 ± 3.83 h (mean ± SD; Table 2) and varied greatly across individuals (coefficient of variation [CV] = 46%) (Supplementary Appendix Figures III–IV).

## DISCUSSION

Our results verify circadian control of singing, namely: (1) the duration of the rhythm has a period of approximately 24 h (entrained conditions: 24.4 ± 0.15 h SE, Figure 2B, and 24.72 ± 0.16 h SE for experiments 1 and 3, respectively, and free-running conditions: 25.1 ± 0.079 h SE, Figure 3A for experiment 2), (2) the rhythm persists (“free-runs”) under constant environmental conditions (Figure 3A–D), (3) the timing of the rhythm is entrained by a Zeitgeber (Figure 4A–C), and (4) the pace of the clock is unaffected by a biologically realistic range of temperatures (“temperature compensation,” Figure 3A). Further, we find no evidence for an influence of temperature on phase markers under entrained conditions (Figure 5, Table 1), though each phase marker advanced upon phase shift by ~1–2 h (Table 2). We also found that, as expected, crickets sing overwhelmingly during the dark phase (Figure 2A). Paired with our quantification of between-individual variation in the timing of phase markers (i.e., onset, peak, and offset) and the marked variation in singing effort, these results highlight circadian singing rhythms as a potential target for both natural and sexual selection (Westwood et al. 2019). Understanding whether singing is controlled by an intrinsic circadian clock versus plastically responsive to environmental stimuli can help inform the evolutionary consequences of a shift in rhythms, or whether a shift in singing rhythms is even possible. For example, responses to selection on timing may be influenced by how clock outputs are translated to initiate singing and if the phase of singing is linked to other circadian traits.

We found that individuals vary in the quantity of time spent singing and in the timing of their rhythms (CV = 46%; Table 2). Further, post hoc analyses reveal a moderate positive correlation between peak phase and singing effort (*r *= 0.53, *P* = 0.044), indicating that the further into the dark phase an individual peaks in singing, the greater their overall singing effort (see Supplementary Appendix Figure V). While onset was not significantly correlated with singing effort (*r* = −0.41, *P* = 0.13; Supplementary Appendix Figure V), it did show a slightly negative trend, whereas offset showed a slightly positive trend (*r* = 0.37, *P* = 0.2; Supplementary Appendix Figure V), possibly suggesting that a wider singing window (i.e., earlier onset and later offset) results in greater singing effort per day. This, coupled with the significant positive relationship between peak and onset, could suggest crickets experience a “warming up” period at the onset of singing (as found in bush crickets and katydids) (Josephson and Halverson 1971; Heller 1986).

Although the laboratory stock population was maintained at a high number of breeding individuals (~100) at any given time, there exists a possibility of laboratory inbreeding. Our identification of circadian clock control of singing is unlikely to have been affected by this, as the de novo evolution of a circadian clock for singing in laboratory conditions which is not present in nature represents a highly un-parsimonious scenario. Inbreeding might be expected to reduce genetic variation in circadian clock control, though we note this is also not supported by our finding of interindividual variation in circadian singing rhythms. If anything, laboratory breeding effects of our stock crickets may have deflated estimates of individual variation. Future studies would benefit from more carefully quantifying the heritability of circadian control of singing in wild populations to establish the evolutionary potential of this trait.

Both normal-wing (singing) and flatwing (silent) males exhibit satellite behavior in this species (i.e., behavior in which non-calling males intercept females attracted to calling males; Zuk, Rotenberry, and Tinghitella 2006; Zuk et al. 2018). As such, variation in singing effort between individuals may be indicative of an individual’s propensity toward satellite tendencies versus commitment to singing. However, juveniles reared in conditions mimicking populations with high levels of singing males are less likely to exhibit satellite behavior (Bailey et al. 2010), and since our population contains ~50% singing males, satellite behavior may not be as prevalent in our population as others showing very high proportions of flatwings (e.g., in Kauai, where upwards of 90% of males are flatwing) (Zuk et al. 2018). However, because singing is energetically costly (Prestwich and Walker 1981; Hoback and Wagner 1997; Hack 1998) and condition-dependent (Holzer et al. 2003; Hunt et al. 2004; Judge et al. 2008; Houslay et al. 2017), individual variation may simply be a result of rearing environment, physiological condition, and/or stochastic developmental trajectories. Interestingly, our observed mean nightly singing effort was seemingly higher than previously reported for Hawaiian *T. oceanicus* (Kolluru 1999). However, Kolluru (1999) removed adult male crickets from the field and observed their singing in the laboratory under ambient lighting conditions, and thus differences in singing effort may be due to the likely poorer condition of wild crickets or disturbances from the data collection methods. Further, while the wild crickets collected by Kolluru (1999) were not age-controlled, all of the crickets in our experiments were placed into experimental conditions within 1–3 days of eclosion and so singing effort may reduce as individuals age.

We found that, in general, male *T. oceanicus* sing between ~ZT13 and ~ZT23, peaking ~ZT17.5. Our results support and develop those of Zuk et al. (1993) who observed that wild *T. oceanicus* sing primarily during the dark phase in the Hawaiian Islands. Further, they found that unparasitised *T. oceanicus* populations begin to sing earlier and continue singing later (i.e., they appear to have a wider singing window) than do the Hawaiian populations (Zuk et al. 1993). Crickets in our experiment rarely sang during the light phase (e.g., crickets in experiment 1 sang only during ~1% of the light phase whereas they sang ~68% of the dark phase), fitting with the notion that selection may have acted on singing rhythms such that individuals in parasitized populations reduce (or, have nearly eliminated) singing at “risky” times-of-day. Future work comparing these two populations from a circadian framework could elucidate the extent to which selection has resulted in temporally distinct circadian singing patterns.

We reveal that nocturnal singing is not simply a phenotypically plastic response to dusk/darkness, but is scheduled by an endogenous circadian clock. Clocks give their owners the ability to anticipate when day/night will occur and so, prepare in advance (Aschoff 1965). Anticipating night-time could be useful for coordinating rhythmic mating behaviors between males and females (Loher and Orsak 1985) or for timing conspicuous singing behavior when parasitism and/or predation risk is low (Zuk et al. 1993). Interestingly, mean onset (~ZT13) occurs about an hour past the start of the dark phase (ZT12)—a finding apparently in contrast with the anticipatory nature of circadian rhythms (though, in line with previous findings in the wild [Zuk et al. 2018]). However, as our lighting system was either on or off (i.e., did not gradually change to mimic dawn and dusk), nuance in anticipation may have been missed. Another possibility is that anticipatory activities occur in advance of the onset of singing, such as a warm-up period or spermatophore production (Josephson and Halverson 1971; Loher 1974; Heller 1986). Further work could ramp light intensity up and down to mimic dawn and dusk to pinpoint the relationships between onset and offset with dusk and dawn, and across the suite of reproductive behaviors crickets engage in.

Singing rhythms appear robust to a range of temperatures under free-running conditions (Figure 3), and entrained conditions (Table 1, Figure 5), though we did find slight evidence for a modest phase advance upon photoperiod reversal (Table 2), possibly due to prolonged transient cycles. The variation in temperature we exposed the crickets to (22–28 °C) approximates the annual variation in temperature in Hawaii where monthly temperatures range from a mean low of 22.8 °C to a mean high of 27.4 °C (National Weather Service, National Oceanic and Atmospheric Administration, monthly summarized data [mean min-mean max°C] in Honolulu, HI from 1950 to 2021). Thus, our experiments examining temperature compensation represent ecologically relevant treatments, and suggest crickets regain the appropriate phase relationship to the Zeitgeber regardless of temperature. For some organisms (e.g., *Neurospora*, *Drosophila*, and mice) temperature can act as an additional Zeitgeber to light (Liu et al. 1998; Sidote et al. 1998; Refinetti 2010). Whether this is the case for crickets could be tested by imposing temperature cycles that align with or oppose light dark cycles to parse out the relative contributions, and potential synergies, of light and temperature as Zeitgebers. Understanding how multiple Zeitgebers operate informs how organisms respond to, for example, climate change, especially in the face of additional selection pressures imposed by infection.

To characterize rhythms from continuous audio recordings, a vast quantity of data are generated that precludes manual scoring. Therefore, we also present a novel audio-to-circadian analysis pipeline, capable of extracting useful parameters from which to train machine learning algorithms, which can then process large quantities of data. Rather than developing a de novo tool, we designed a modular and open-source R package, Tempaural (https://github.com/rethomics/tempaural/), as an add-on to the Rethomics framework (Geissmann et al. 2019). Tempaural handles the conversion of multiple audio files into standardized meta-variables and arbitrary acoustic (i.e., behavioral) variables. Within the documented Rethomics framework, we can then readily visualize behavior states (singing) over time and, for instance, compute circadian statistics. The application of machine learning techniques toward bioacoustic analysis is gaining traction (Aide et al. 2013; Zhang et al. 2017) and our pipeline can be used for any sound-producing species, whether for circadian analysis or simply for detecting signal in noise. Further, the pipeline may be applied to organisms not typically considered to acoustically advertise, including the detection of vibrational signals recorded on contact microphones. This could be especially useful in investigating singing rhythms in flatwing males, who do not produce song per se (Schneider et al. 2018), but have been shown to exhibit singing effort similar to normal-wing males (Rayner et al. 2020). However, whether flatwing males stridulate consistently throughout the night and/or maintain the same phase relationship with light as do normal-wing males remains unresolved.

In summary, we demonstrate that singing rhythms in *T. oceanicus* meet all four requirements necessary to be deemed under the control of an endogenous circadian oscillator. Our findings are largely in agreement with past efforts toward elucidating the timing of singing (Loher and Orsak 1985; Zuk et al. 1993; Kolluru 1999), with some interesting differences in observed singing effort. Our work adds to this literature by interrogating singing from a robust circadian framework, which is important to show that the phase relationship of a behavior (upon which selection is likely to act) is indeed heritable and not simply a plastic response (i.e., a reactionary or “just in time” response) to the environment.

## Supplementary Material

arad098_suppl_Supplementary_Material

## Data Availability

Analyses reported in this article can be reproduced using the data provided by Westwood (2023).
