# Supplementary material for: Machine learning reveals singing rhythms of male Pacific field crickets are clock controlled
Source: Behav Ecol. 2023 Dec 23;35(1):arad098. doi: 10.1093/beheco/arad098 (PMC10748470; doi:10.1093/beheco/arad098)
Supplement: arad098_suppl_Supplementary_Material [file arad098_suppl_supplementary_material.zip › Appendix.docx]

**Appendix**

| Parameter | Definition |
| --- | --- |
| freq.M | Median frequency |
| freq.IPR | Frequency interpercentile range |
| spec.mean | Mean of the frequency in the 3-6 kHz spectrum |
| spec.median | Median of the frequency in the 3-6 kHz spectrum |
| spec.mode | Mode of the frequency in the 3-6 kHz spectrum |
| freq.P2 | Frequency terminal percentile |
| time.M | Time median |
| freq.P1 | Frequency initial percentile |
| spec.sd | S.D. in the 3-6 kHz spectrum |
| spec.sem | S.E.M. in the 3-6 kHz spectrum |
| spec.Q25 | First quartile in the 3-6 kHz spectrum |
| spec.Q75 | Terminal quartile in the 3-6 kHz spectrum |
| spec.IQR | Interquartile range in the 3-6 kHz spectrum |
| spec.cent | Centroid in the 3-6 kHz spectrum |
| spec.skewness | Skewness in the 3-6 kHz spectrum |
| spec.kurtosis | Kurtosis in the 3-6 kHz spectrum |
| spec.sfm | Spectral flatness measure in the 3-6 kHz spectrum |
| spec.sh | Spectral entropy in the 3-6 kHz spectrum |
| spec.prec | Frequency precision of the 3-6 kHz spectrum |

**Table I.** Parameters derived from audio clips using the Seewave R package. Parameters highlighted in yellow were chosen to train the models.

|  | Reference | | |
| --- | --- | --- | --- |
| Prediction |  | n | y |
|  | n | 114 | 2 |
|  | y | 0 | 22 |

**Table II.** Random forest model confusion matrix. Model “predictions” (“n” = no, “y” = yes, pertaining to whether a given clip contained chirping or not) on the left are compared against known “reference” clip values (“n” = no, “y” = yes along the top). The model predicted “no” correctly 114 times and incorrectly 2 times. The model predicted “yes” correctly 22 times, and “yes” incorrectly 0 times. Thus, the model (though overall very accurate, 98% as shown in Table III) is more likely to supply false negatives than false positives, rendering the model both highly accurate and conservative.

| Accuracy | 0.9855 |
| --- | --- |
| 95% CI | (0.9486, 0.9982) |
| No Information Rate | 0.8261 |
| P-Value | 1.592e-09 |
| Kappa | 0.9478 |
| Mcnemars Test P-value | 0.4795 |
| Sensitivity | 1.0000 |
| Specificity | 0.9167 |
| Pos Pred Value | 0.9828 |
| Neg Pred Value | 1.0000 |
| Prevalence | 0.8261 |
| Detection Rate | 0.8261 |
| Detection Prevalence | 0.8406 |
| Balance Accuracy | 0.9583 |

**Table III.** Accuracy statistics associated with the random forest model.

**Figure I.** Accuracy and kappa results from three algorithms (rf=random forest, cart=classification and regression tree, kNN=k-nearest neighbors) tested using k-fold cross validation.

**Figure II.** Experiment 2: Individual Lomb-Scargle periodograms with most significant period estimate indicated by the black x on each plot. Temperature treatment groups are indicated by colours in the legend (22°C=blue, 25°C=purple, and 28°C=pink). Power of a given period estimate is located on the y-axis, and period (in hours) is located on the x-axis.

**Figure III.** Experiment 3: Individual Lomb-Scargle periodograms during the LD lighting regime with most significant period estimate indicated by the black x on each plot. Temperature treatment groups are indicated by colours in the legend (22°C=blue, 25°C=purple, and 28°C=pink). Power of a given period estimate is located on the y-axis, and period (in hours) is located on the x-axis.

**Figure IV.** Experiment 3: Individual Lomb-Scargle periodograms during the DL lighting regime with most significant period estimate indicated by the black x on each plot. Temperature treatment groups are indicated by colours in the legend (22°C=blue, 25°C=purple, and 28°C=pink). Power of a given period estimate is located on the y-axis, and period (in hours) is located on the x-axis.

**Figure V.** Correlation plots for each phase marker (**A.** Onset, **B.** Peak, and **C.** Offset; x-axes) in ZT and “singing effort” in hours (y-axes). Spearman’s correlation coefficient (*R*) and associated p-values are shown on each plot, along with points representing individuals, the regression line, and shaded areas are the confidence interval.
