# Supplementary figures and images for: Machine learning reveals singing rhythms of male Pacific field crickets are clock controlled
Source: Behav Ecol. 2023 Dec 23;35(1):arad098. doi: 10.1093/beheco/arad098 (PMC10748470; doi:10.1093/beheco/arad098)

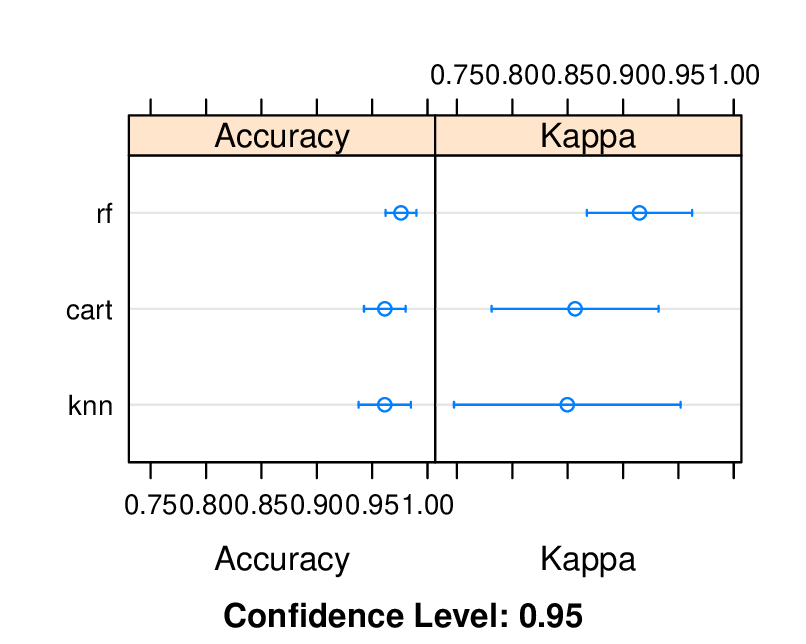

Supplement: arad098_suppl_Supplementary_Material [file arad098_suppl_supplementary_material.zip › Figure_I.tiff]

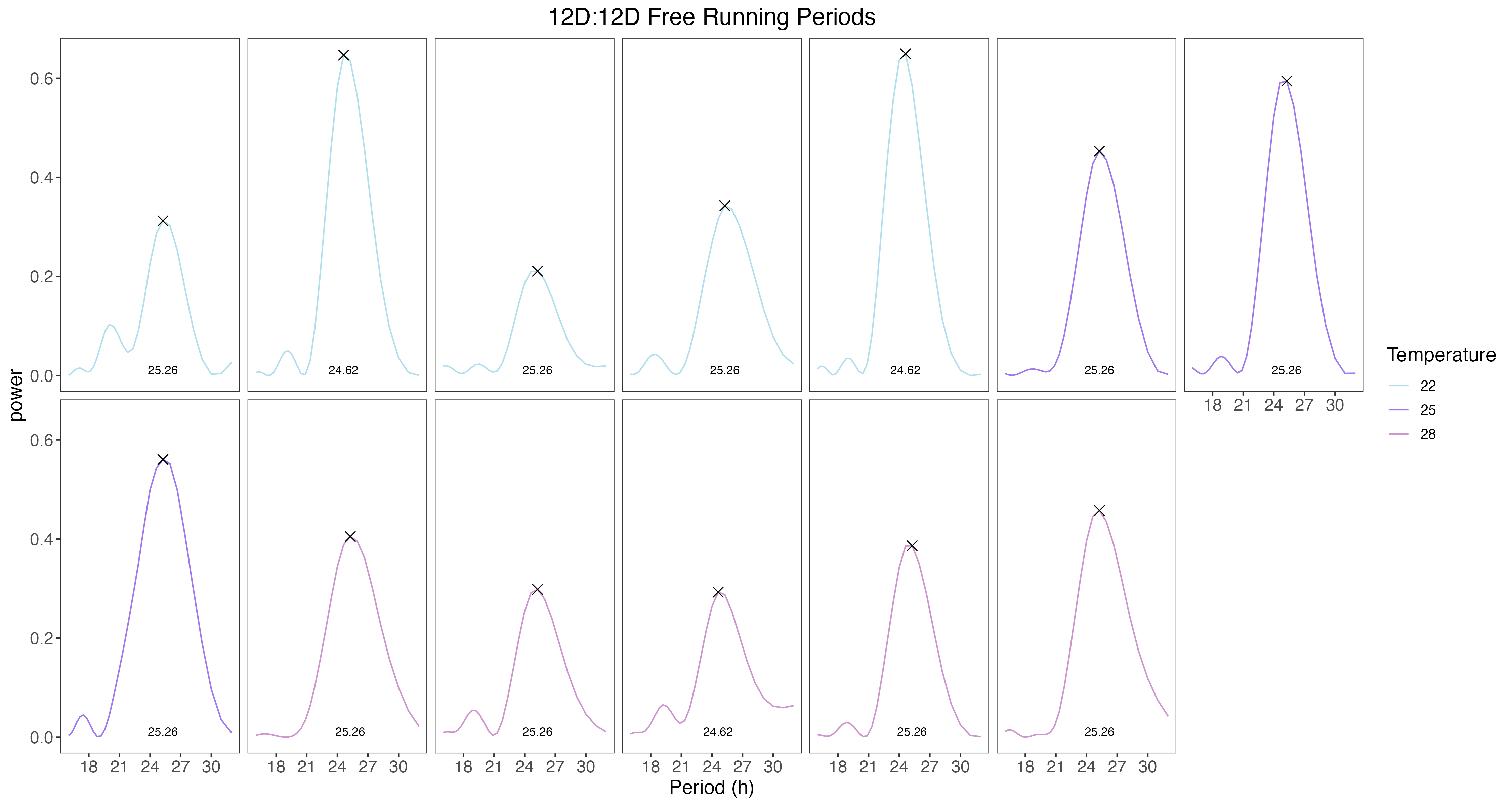

Supplement: arad098_suppl_Supplementary_Material [file arad098_suppl_supplementary_material.zip › Figure_II.tiff]

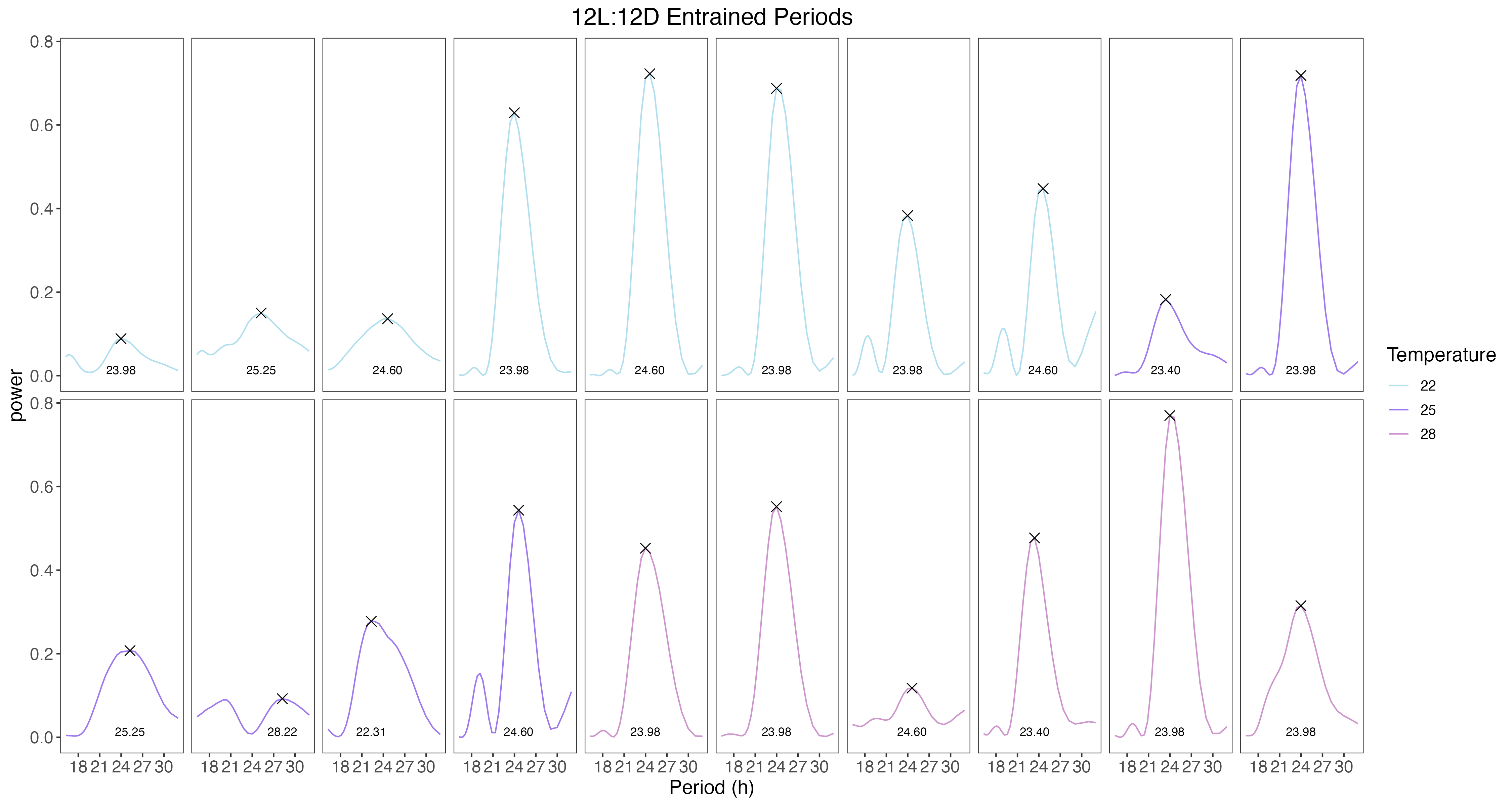

Supplement: arad098_suppl_Supplementary_Material [file arad098_suppl_supplementary_material.zip › Figure_III.tiff]

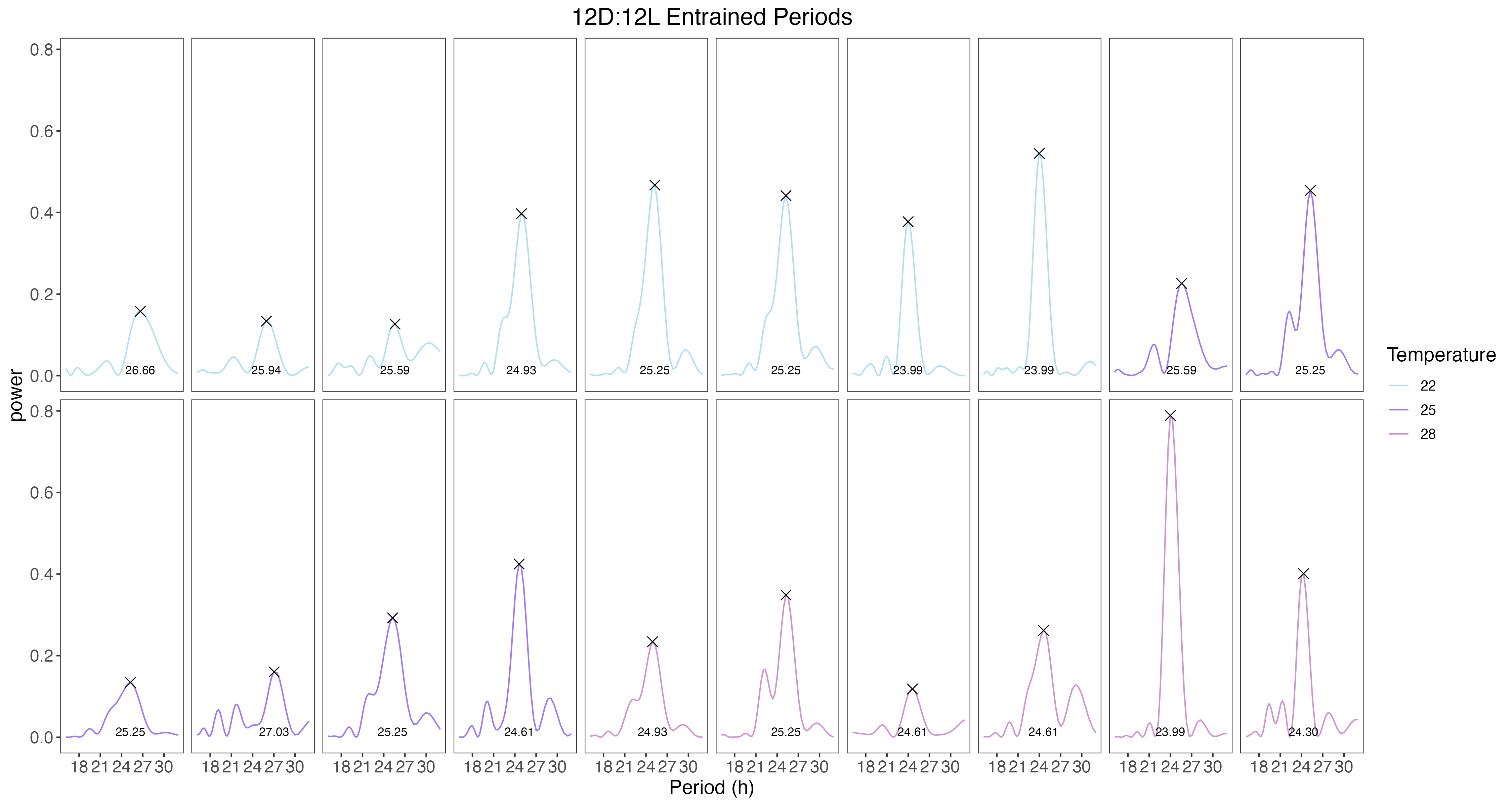

Supplement: arad098_suppl_Supplementary_Material [file arad098_suppl_supplementary_material.zip › Figure_IV.tiff]
